# Evaluation of Antioxidant Capacity (ABTS and CUPRAC) and Total Phenolic Content (Folin-Ciocalteu) Assays of Selected Fruit, Vegetables, and Spices

**DOI:** 10.1155/2022/2581470

**Published:** 2022-11-30

**Authors:** Jenson George, David Edwards, Sharon Pun, David Williams

**Affiliations:** Department of Agriculture and Fisheries, Health and Food Sciences Precinct, Block 10, 39 Kessels Road, PO Box 156, Archerfield BC, Coopers Plains, QLD 4108, Australia

## Abstract

Antioxidant (AOX) capacity assays are important analytical tools, used worldwide to measure the AOX capacities of various food commodities. Although numerous protocols have been published to ascertain AOX capacities, there are increasing concerns about the reliability of many of these assays. Poor correlation of results between various assays, as well as problems with reproducibility, consistency, and accuracy, is to blame. Published AOX assays also differ markedly from each other by employing different reaction conditions, using different extracting solvents, and applying dissimilar quantification methods. In this study, AOX capacities of a range of fruit, vegetables, and spices, commonly consumed and of commercial importance in Australia and worldwide, were measured in both hydrophilic and lipophilic solvents by using two different assay systems. As the polyphenolic compounds present in any sample matrix are the main contributors to its AOX properties, the commodities were also analysed for total phenolic content (TPC), again using both solvent systems. Analysis of the results from the current study with values from the published literature exposed the challenges that make direct comparison of any quantitative results difficult. However, a strong mutual correlation of our assay results facilitated a meaningful comparison of the data within the laboratory. Concurrent use of lipophilic and hydrophilic solvents made the results more reliable and understandable. Findings from this study will aid to address the existing challenges and bring a more rational basis to the AOX capacities. This unique analytical approach also provided a platform to build an internal reference database for the commonly consumed and commercially important food commodities with the potential to broaden the scope into a database for similar food matrices.

## 1. Introduction

Antioxidants (AOXs), the molecules that fight against harmful free radicals and protect cells, are considered essential for the survival of all living things. This in turn has led to a rising awareness within the scientific community of the need to develop simple but effective assays that measure the antioxidant (AOX) properties of foods. AOX capacity assays are important analytical tools, used worldwide to measure the AOX capacities of various food commodities. In principle, the assay involves a chemical reaction which allows AOXs present in the sample to react with a set concentration of the assay reagent. The course of the reaction is monitored and the consumption of the assay reagent by the sample extract is measured instrumentally. The subsequent readings can then be interpreted as AOX capacity values. Though the procedure appears simple in principle, most assays involve many complex and diverse reaction mechanisms [1, 2]. One of the main challenges in the development of a universal AOX capacity assay is that, within any sample matrix, there are numerous AOXs, which can react with a particular assay reagent in different ways based on their molecular structures and physicochemical properties [1]. Examples of chemical reactions between AOXs and an assay reagent are hydrogen atom transfer (HAT), single electron transfer (SET), and the chelation with transition metals, which are largely influenced by assay reaction conditions such as time, pH, temperature, and reaction medium (solvent) [1–3].

To study the AOX capacity of the commonly consumed fruit, vegetable, and spices, the commonly employed AOX assay systems are Oxygen Radical Absorbance Capacity (ORAC), Total Radical Trapping AOX Parameter (TRAP), 2,2-diphenyl-1-picrylhydrazyl (DPPH), Vitamin C Equivalent AOX Capacity (VCEAC), 2,2′-azino-bis (3-ethylbenzothiazoline-6-sulfonic acid) (ABTS), Trolox Equivalent AOX Capacity (TEAC), Ferric Ion Reducing AOX Power (FRAP), and Cupric Ion Reducing AOX Capacity (CUPRAC). Having a specific or combined AOX target within a sample matrix, each AOX assay system exhibits its own distinct advantages and disadvantages [1, 2]. Clearly, matching assay reagent and system characteristics to the AOX reaction mechanisms are critical in the selection of appropriate AOX assay methods. Furthermore, AOX compounds present in food-based matrices can be either water-soluble (hydrophilic) or fat-soluble (lipophilic) [2–4]. So, it is very important that the selected assay can incorporate the determination of both the lipophilic and hydrophilic components if a meaningful assessment of the total AOX capacity is to be made. Because multiple reaction mechanisms are usually involved, no single assay will accurately reflect all AOXs in a mixed or complex sample. It must be understood from the outset that no two assays will necessarily produce the same value for a given AOX compound [5] and that there is no simple universal method by which AOX capacity can be measured accurately and quantitatively [1]. It is difficult to compare the quantitative results obtained by different AOX assay methods because of the diverse range of mechanisms, solvents used, and reaction conditions of the various assays [2]. Compounding the issue is the expression of results in different units (*μ*mol, mmol, or mg of the reference standard equivalent per g, 100 g, or kg of the sample on a fresh or dry weight basis). Since there is no single universal method to unequivocally measure AOX capacities, the technical report of International Union of Pure and Applied Chemistry (IUPAC) [2] highly recommends measuring the AOX capacity of any given sample by two independent assay systems to ensure meaningful comparisons are made within a laboratory.

It is also recommended by most researchers that the total phenolic content (TPC) be ascertained to complement the AOX capacity assay due to the belief that the polyphenolics present in any food-based sample matrix are the main contributors to its AOX properties [4, 6]. However, the conventional Folin-Ciocalteu total polyphenolic content (FC-TPC) assay [7] traditionally used to measure the TPC is not compatible with lipophilic polyphenolic molecules because increased concentrations of lipophilic extracting solvents like ethanol can lead to the precipitation of the assay reagent [3, 8, 9]. Thus, the use of hydrophilic solvents alone can result in the exclusion of lipophilic polyphenols and their contribution in the TPC assay.

Assay systems addressing the above-mentioned shortcomings in the application of the AOX capacity and TPC assays were not available at the time of the current study. It is essential to have reliable methods established in any analytical laboratory to utilise these useful assays to produce meaningful results. It is also important to establish a reference database for a wide range of sample matrices to make meaningful comparisons.

Therefore, the aim of this work was to overcome the shortcomings in applying the AOX capacity and TPC assays, to address challenges that prevent meaningful comparisons with the published values, and to apply the improved assay conditions to a range of fruit, vegetables, and spices commonly consumed in Australia and worldwide (sample details are given under Section 2.1). The authors of this study chose a modified ABTS assay by Ozgen et al. [10] with assay reaction conditions at pH 4.5 (this has the advantage of increased stability for the assay reagents as well as the AOXs and ascorbic acid (AA) reference standards), a CUPRAC assay based on Apak et al. [11] (which has the advantages of better reagent stability than radical reagents like ABTS, a neutral pH of 7.0 that is close to the biological systems, and compatibility towards hydrophilic and lipophilic solvents), and a modified FC-TPC assay based on Pereira et al. [12] with reaction conditions at pH 11.5 (which has the benefit of better compatibility towards lipophilic solvent systems than the conventional method by Singleton and Rossi [7]). Ultrapure (Milli-Q) water was selected as the hydrophilic extracting solvent, whereas 40% ethanol in Milli-Q water was employed as the lipophilic extracting solvent. Suitability of the selected assays within our laboratory conditions was investigated. Using the selected methodologies and solvent systems, the AOX capacity and TPC of fruit, vegetables, and spices were evaluated. The scope of expansion of results to a reference database was also investigated.

## 2. Materials and Methods

### 2.1. Materials

The samples (collected between October 2020 and January 2021) consisted of (1) fruit–duplicate punnets of blueberries (CO_OP Ltd, OzGroup), blackberries (Driscoll's), raspberries (the berry collective raspberries), and cherries (Cherry Isle, Tasmania PLU, 4045) purchased from various supermarkets in Brisbane, Queensland, Australia; pale and red coloured strawberry varieties (identified as MRF, 2020, 217-230 and MRF, 2020, 2017-139, respectively) from Maroochy Research Facility (MRF) of the Queensland Department of Agriculture and Fisheries (DAF); a plum variety (identified as ARF, 2020, 401-43, a relative of Queen Garnet plum) from Applethorpe Research Facility (ARF) of DAF; and a commercial sample of Queen Garnet plum freeze-dried powder from Nutrafruit Pty Ltd, Australia (purchased in June 2018); (2) fruit waste–papaya seeds separated from fruit purchased from a local supermarket and pomegranate seeds and husks separated from fruit (POM and SunnyGem, 3127 USA) purchased from a local supermarket; (3) vegetables–green, yellow, and red capsicums (Australian Mini *Capsicums*) purchased in duplicate from various local supermarkets; pumpkin (Kent cut, Australian Grown) purchased in duplicate from local supermarkets; and fresh whole tomatoes purchased from local markets; and (4) spices–turmeric powder, ginger powder, cocoa powder, and green tea (Twinings of London) bags, all purchased from various local stores. Specific information about the varieties of fruit, vegetables, and spices sourced from supermarkets was not available.

Analytical grade reference standards of L-ascorbic acid (AA), gallic acid (GA), and 6-hydroxy-2,5,7,8-tetramethylchroman-2-carboxylic acid (Trolox) were purchased from Sigma-Aldrich, Australia. All other reagents and chemicals used (copper (II) chloride dihydrate, neocuproine, ABTS, potassium persulfate, methyl-beta-cyclodextrin (m*β*CD), sodium acetate, ammonium acetate, Folin-Ciocalteu (FC) reagent, sodium carbonate, and ethanol) were of high-quality analytical grade obtained from Sigma-Aldrich, Australia, and the water used was ultrapure Milli-Q from Merck-Millipore.

### 2.2. Methods

#### 2.2.1. Sample Preparation

Samples were prepared for extraction based on the collective information gathered from multiple references listed in Table 1. The edible portions of the berries, capsicum, cherry, plum, and pumpkin were separately hand chopped into small pieces, freeze-dried for seven days, and finally milled in a ball mill (Retsch MM400) to get uniform powders. Samples of papaya seeds, pomegranate seeds, and husks were separately dried in an oven (Gallenkamp, Oven 300) at 40°C for 24 hours and milled in a ball mill (Retsch MM400) to get uniform powders. Green tea, turmeric powder, ginger powder, cocoa powder, and commercial Queen Garnet plum freeze-dried powder were used as received in commercial packaging. Fresh whole tomatoes were pureed using a domestic blender to get a consistent sample. All the resulting samples were quickly transferred to 50 mL centrifuge tubes and stored at -80°C till the time of analysis.

#### 2.2.2. Moisture Content

The moisture content of the prepared samples was determined from the weight loss after drying to constant weight at 70°C under vacuum (AOAC) [13]. Duplicates of approximately 0.2 g of the samples (except 2.0 g for fresh tomato) were tested in this manner. The percentage difference in weight loss after drying was expressed as the moisture content in g/100 g sample.

#### 2.2.3. Sample Extraction

Samples were extracted based on the collective information gathered from multiple references listed in Table 1. Milli-Q water was selected as the hydrophilic extracting solvent, whereas 40% ethanol in Milli-Q water was employed as the lipophilic extracting solvent. m*β*CD is used as a solubility enhancer to bring hydrophilic and lipophilic components together.

Triplicates of known weights of the prepared samples were separately extracted in different solvent systems that were proven to be suitable for the hydrophilic and lipophilic components present in the samples and compatible with the three assay systems.

Hydrophilic solvent extraction (marked as Milli-Q water for solvents in tables): approximately 0.2 g of sample was accurately weighed into a 15 mL centrifuge tube. 10 mL of Milli-Q water was added and the tubes were vortexed for 8-10 seconds for homogenous mixing. The resulting mixture was sonicated for 10 min with occasional manual shaking and centrifuged for 5 min at 4000 rpm at 20°C

Lipophilic solvent extraction (marked as 40% ethanol for solvents in tables): approximately 0.2 g of sample was accurately weighed into a 15 mL centrifuge tube. 10 mL of 40% ethanol was added and the tubes were vortexed for 8-10 seconds for homogenous mixing. The resulting mixture was sonicated for 10 min with occasional manual shaking and centrifuged for 5 minutes at 4000 rpm at 20°C

Extracts in the presence of methyl-beta-cyclodextrin (marked as m*β*CD for solvents in Tables 2 and 3): the lipophilic extract in 40% ethanol was modified by diluting it 1 : 1 with 7% *w*/*v* m*β*CD in Milli-Q water. m*β*CD is used as a solubility enhancer to bring hydrophilic and lipophilic components together

Similarly, triplicate 0.2 g samples of the commodities that contain carotenoids (capsicum, tomato, and pumpkin) were separately extracted in both 100% ethanol and dichloromethane due to their high hydrophobic nature.

All the sample extracts were further diluted with matching solvents to obtain acceptable absorbance values that fell within the reference standard concentration range of the respective assays.

#### 2.2.4. Assay Conditions

*(1) ABTS Assay*. The assay was based on Ozgen et al. [10]. To a disposable glass tube was added 1 mL of the ABTS working solution and allowed to stabilise at 28°C in a water bath for 10 min. 50 *μ*L of diluted sample extract or AA standard solution (freshly prepared in Milli-Q water immediately before the addition, with concentrations ranging from 100 *μ*M to 700 *μ*M) was added to the tube, vortexed, and allowed to incubate for 60 min at 28°C. The absorbance at 734 nm was recorded using a Shimadzu UV 1280 spectrophotometer (Shimadzu, Australia). A standard calibration curve was constructed by plotting absorbance versus the concentration of the AA standards. The AOX capacity of the sample was calculated from the slope of the calibration curve using equations (1) and (2) and expressed as *μ*mol AA equivalents (AAE)/g of sample.

(1) $$\begin{array}{c} y=\text{m x}+C, \end{array}$$

(2) $$\begin{array}{c} \mu \text{mol of AAE}/\text{g sample}=\frac{x*\text{DF}*\text{Vi}*}{\text{Ws}*1000}, \end{array}$$

where *y* is the absorbance at 734 nm after blank correction, *m* is the gradient of the graph, *x* is the *μ*M AAE per mL of standard or sample in the assay, *C* is the y-intercept of the calibration curve, DF is the dilution factor, Vi is the initial sample volume (mL), and Ws is the weight of sample (g).

The parameter estimates of “*m*” and “*C*” were obtained from the standard calibration curve, and 1000 is used for M to mol conversion.

The stability of the ABTS assay reagents and the assay incubation time were verified in our laboratory by (1) measuring the absorbance of the ABTS working solution at 734 nm before and after assay procedures, (2) monitoring the stability of the range of AA standard solutions over time by measuring their absorbance values, and (3) optimising the assay incubation time by measuring the AOX capacity of a representative sample over time.

*(2) CUPRAC Assay*. The assay was based on Apak et al. [11]. To a 15 mL centrifuge tube were added 1 mL each of 1.0 × 10^−2^ M copper (II) chloride dihydrate, 7.5 × 10^−3^ M neocuproine, and 1 M pH 7.0 ammonium acetate buffer solution. A known volume (x mL) of diluted sample extract or Trolox standard solution (prepared in 96% ethanol with concentrations ranging from 100 *μ*M to 1200 *μ*M) and solvent (Milli-Q water or 40% ethanol (1.1–x mL)) were added to the initial mixture to make the final volume 4.1 mL. The tubes were vortexed for homogenous mixing and incubated for 30 min at ambient temperature. The absorbance at 450 nm was recorded against a reagent blank using a Shimadzu UV 1280 spectrophotometer. A standard calibration curve was constructed by plotting absorbance versus the concentration of the Trolox standards in the assay and the molar absorption coefficient determined. The AOX capacity of the sample was calculated using equation (3) and expressed as mmol Trolox equivalents (TE)/g of sample.

(3) $$\begin{array}{c} \text{mmolTE}/\text{gsample}=\frac{\text{Abs}*\text{Vf}*\text{DF}*\text{Vi}}{\varepsilon *\text{Vs}*\text{Ws}}, \end{array}$$

where Abs is the absorbance at 450 nm after blank correction, Vf is the final volume of assay solution (mL), DF is the dilution factor, Vi is the initial sample volume (mL), *ε* is the molar absorption coefficient, calculated from standard calibration curve, Vs is the volume of sample extract used in assay (mL), and Ws is the weight of sample (g).

Assay results obtained for the samples in mmol TE/g were then converted to *μ*mol TE/g by applying unit conversions.

The assay conditions were verified in our laboratory by (1) monitoring the stability of the range of Trolox standard solutions over time by measuring their absorbance values and (2) optimising the assay incubation time based on these results.

*(3) FC-TPC Assay*. The assay was based on Pereira et al. [12]. To a disposable glass tube was added 100 *μ*L of diluted sample extract or GA standard solution (prepared in Milli-Q water or 40% ethanol with concentrations ranging from 50 *μ*g/mL to 250 *μ*g/mL) followed by 100 *μ*L of diluted FC reagent (diluted 1 : 1 in Milli-Q water) and 800 *μ*L of 5% sodium carbonate. The tubes were vortexed for consistent mixing and incubated for 20 min at ambient temperature. The absorbance at 750 nm was recorded using a Shimadzu UV 1280 spectrophotometer. A standard calibration curve was constructed by plotting absorbance versus the concentration of the GA standards. The concentration of the unknown AOX was calculated from the slope of the calibration curve using equations (4), (5), and (6) and expressed as mg GA equivalents (GAE)/100 g sample.

(4) $$\begin{array}{c} y=\text{m x}+C, \end{array}$$

(5) $$\begin{array}{c} \mathrm{\mu gGAE}/\text{mLsample in assay }\left(x\right)=\frac{y-C}{m}, \end{array}$$

(6) $$\begin{array}{c} \text{mgGAE}/100\text{gsample}=\frac{x*\text{DF}*\text{V}i}{\text{Ws}*10}, \end{array}$$

where, *y* is the absorbance at 750 nm after blank correction, *m* is the gradient of the graph, *x* is the *μ*g GAE per mL of standard or sample in the assay, *C* is the y-intercept of the calibration curve, DF is the dilution factor, Vi is the initial sample volume (mL), and Ws is the weight of sample (g).

The parameter estimates of “*m*” and “*C*” were obtained from the standard calibration curve, and 10 is the correction factor to convert the results in *μ*g to mg and adjusted for 100 g.

Assay results obtained for the samples in mg GAE/100 g are then converted to *μ*mol GAE/g by taking the molecular weight of GA into account and by applying unit conversions.

The FC-TPC assay conditions were verified in our laboratory by (1) monitoring the stability of the GA standard solutions in 40% ethanol at two separate time points by measuring their absorbance values, (2) monitoring the behaviour of the GA standards in two different solvent systems (Milli-Q water and 40% ethanol) at two different absorption wavelengths (750 nm and 760 nm), and (3) optimising the incubation time by measuring the absorbances of the range of GA standards at different time points.

## 3. Results and Discussion

### 3.1. Moisture Content

The moisture contents of the prepared samples which were either fresh, freeze-dried, or oven-dried are given in Table 4. The moisture results were used to convert the results of the individual assays (from equations (2), (3), and (6)) to dry weight basis facilitating comparison with the published literature values for similar food products.

### 3.2. Verification of Assay Conditions

#### 3.2.1. ABTS Assay Verification

Absorbance of the ABTS working solution at 734 nm was monitored before and after assay procedures and recorded as absorbance units (AU): beginning of the experiment: absorbance of working ABTS = 0.999 AU and end of the experiment: absorbance of working ABTS = 0.998 AU.

The results indicated that the ABTS working solution (in pH 4.5 acetate buffer) was stable (without any decomposition) throughout the assay eliminating the chances of absorbance measurement inaccuracies due to breakdown of the ABTS.

**Figure 1:**
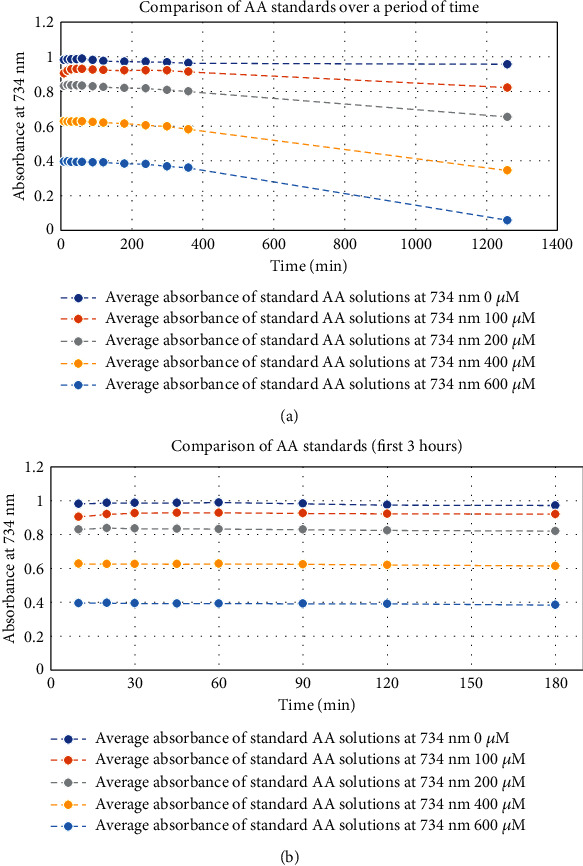
(a) Stability of AA working standards over time. (b) Stability of AA working standards for the first 3 hours.

Stability of the AA standards was monitored over a range of concentrations (in triplicate) by measuring their absorbance values and plotting their average against a range of time points (see Figure 1(a)). A close examination of the values confirmed that the working AA standards were stable for the first three hours of the assay (see Figure 1(b)), thereby avoiding absorbance measurement inaccuracies due to the decomposition of standards in the assay system.

**Figure 2:**
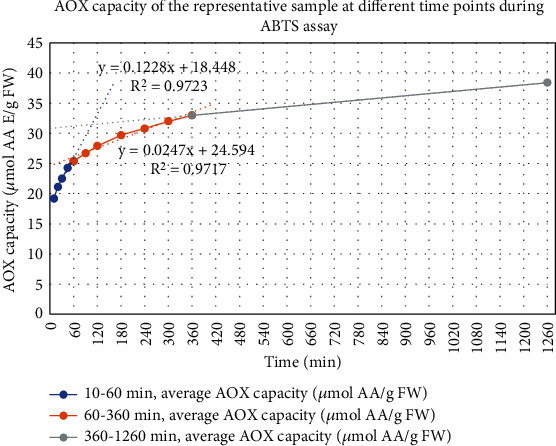
AOX capacities of the same sample at different incubation time points.

The assay incubation time was optimised based on the graphical plot of the AOX capacity results for a representative sample obtained at a range of time points. Tangents were drawn to predict the linearity of the graph at 3 different sets of time points based on their linear trend in the first 60 minutes (min), the next 5 hours, and from there to the next 15 hours (see Figure 2).

The assay tends to a steady state after 60 min, in line with the findings of Ozgen et al. [10]. Consequently, the optimal incubation time was set at 60 min.

#### 3.2.2. CUPRAC Assay Verification

**Figure 3:**
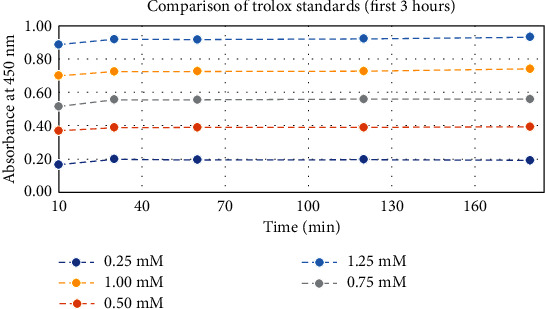
Stability of Trolox working standards over time.

Stability of the Trolox standards was monitored over a range of concentrations (in triplicate) by measuring their absorbance values and plotting their average against a range of time points (see Figure 3).

It was confirmed that the working Trolox standards were stable for the first three hours of the assay (see Figure 3), thereby removing any absorbance measurement inaccuracies due to the decomposition of standards in the assay system. As evident from Figure 3, the assay reaches a stable value after 30 min and remains stable for the first three hours. So, 30 min was selected as the assay incubation time, agreeing with that reported by Gulcin [6].

#### 3.2.3. FC-TPC Assay Verification

**Figure 4:**
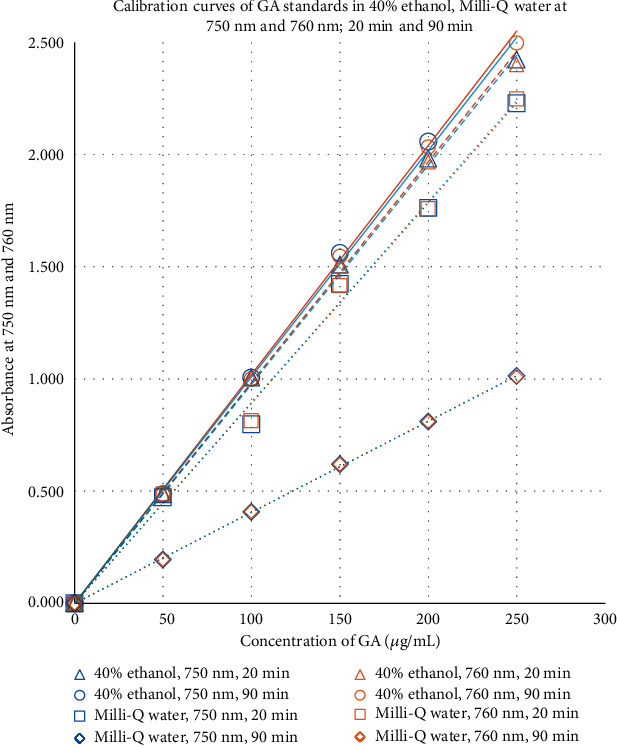
Calibration curves of GA standards in 40% ethanol and Milli-Q water, at 750 nm and 760 nm, after 20 min and 90 min of incubation.

Absorbances of GA standard solutions with concentrations (in triplicate) ranging from 0 to 250 *μ*g/mL, in 40% ethanol and in Milli-Q water, were separately measured after 20 min and 90 min of incubation and at two wavelengths, 750 nm and 760 nm. The calibration curves obtained by plotting their respective average absorbance values against concentration (*μ*g/mL) of GA standard solutions are given in Figure 4.

The verification steps confirmed that the assay reagent and GA standards used in the current study were stable in 40% ethanol, as stipulated in the modified assay by Pereira et al. [12], as well as Milli-Q water used in the conventional method by Singleton and Rossi [7]. The mentioned verification also confirmed the suitability of a shorter assay incubation time of 20 min when compared to 90 min used in the conventional method. The comparability of the two different wavelengths of absorption reported [7, 12], i.e., 750 nm and 760 nm (Figure 4 and Table 5), was also noted. Due to the reliability of the shorter assay incubation time and similarity of the absorbance readings in both the reported wavelengths of absorption, a 20 min assay time and 750 nm were selected as the most appropriate conditions for consistency.

**Figure 5:**
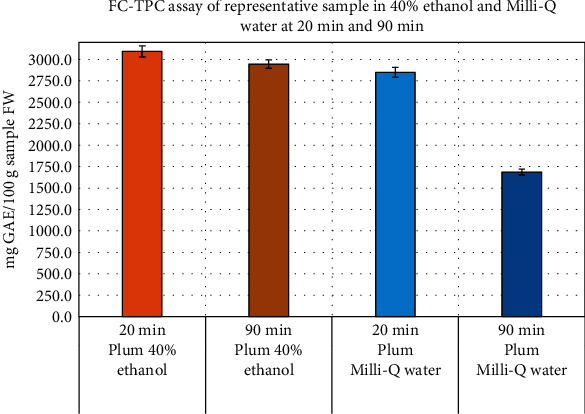
FC-TPC assay of a representative sample in 40% ethanol and Milli-Q water at 20 and 90 min.

A representative sample was assayed using both TPC methods [7, 12] and the results were compared (see Figure 5). It was confirmed from the results that the conventional hydrophilic extract in Milli-Q water produced much lower TPC assay results compared to the lipophilic extracts using 40% ethanol, presumably as the hydrophilic solvent was not able to extract the lipophilic polyphenols present in the sample [2–4, 6]. The standard calibration curve of pure gallic acid standard in Milli-Q water at 90 min was found to be different from that of at 20 min (Figure 4) possibly due to stability issues of such polyphenolic compounds at a higher pH over time. Thus, both the hydrophilic and lipophilic extraction solvents were selected for our studies and the results compared.

### 3.3. Assay Results—Comparison with Reported Values

The results from the current study are provided in Table 2 and are expressed as *μ*mol of reference standard equivalents per gram on a dry weight basis.

Direct comparisons were not possible between the results of our study and most of the previously published assay values due to the vast differences in the published literature, in terms of extracting solvents, assay incubation time, selection of different reference standards, presentation of results in different units, etc. (Tables 1 and 2). To facilitate a meaningful comparison, we have converted the units of the published assay values by taking molecular weights and correction factors into account and further applying appropriate correction for the moisture content (based on the data available from United States Department of Agriculture (USDA)–Nutritional Value of Foods, Home and Garden Bulletin No. 72 [52]) to convert from a fresh weight to a dry weight basis if necessary. The published values are also presented in Table 2 (^ɸ^: reported values after unit conversions; ^∗^: published reference).

It became apparent from Table 2 that, though the matching assays show some similarities in their range of results (after converting to similar units), the range of assay results obtained in the current study was not directly comparable with the previously reported values.

In addition to the challenges associated with direct comparison of the assay results in Table 2, it is worth considering other contributors that may make such comparisons inappropriate and problematic. These include agrienvironmental factors such as the varietal differences, ecological influences, growing conditions, degree of ripening, and the natural variation of these compounds in the sample matrix.

Fruit in general, especially berries, are very good sources of phenolic compounds including anthocyanins, ellagic acid, GA, quercetin, ferulic acid, catechin, and caffeic acid [21, 53, 54]. However, notable differences have been reported for TPC and AOX capacities between berry varieties; this is probably due to agrienvironmental factors and the natural variation of these compounds in the fruit [14, 15, 21].

The main reported polyphenolics in capsicums are GA, chlorogenic acid, various glucopyranosides, capsaicin, quercetins, myricetin, and carotenoids [55, 56]. *β*-Carotene, catechin, chlorogenic acid, caffeic acid, coumaric acid, kaempferol, ferulic acid, salicylic acid, and syringic acid are the main reported polyphenols in pumpkins [26, 27]. Various phenolic acids, flavonoids, lycopene, and rutin apioside are reported in tomatoes [22, 28, 57]. Similar to fruit, the levels of phenolic compounds and carotenoids in vegetables are extremely variable, with agrienvironmental factors again contributing. The differences in the concentration of these compounds in vegetables are the key reason for their varying TPC and AOX capacities [22, 26–28, 55–57].

The key polyphenolic compounds reported in papaya seed are various phenolic acids, anthocyanins, quercetins, catechins, GA, phenolic aldehydes, coumarin, and phenolic diterpenes [29, 30]. Pomegranate seed extracts contain catechins, sterols, tocopherols, polyphenolic acids, and fatty acids. Pomegranate husk is rich in ellagitannins, GA, fatty acids, catechin and epicatechin, quercetin, rutin and other flavonols, flavones, flavonones, proanthocyanidins, and anthocyanidins [34]. Very high levels of catechin and GA were detected in the husk compared to other parts of the pomegranate fruit including the seeds and reported to exhibit higher assay results [34, 36]. As for fruit and vegetables, the seeds and peel of fruit vary in their levels of phenolic composition depending upon the genotype, cultivar, varietal differences, etc., and the variation in the concentration of these compounds results in their varying TPC and AOX capacities.

The major bioactive constituents reported in turmeric samples are diarylheptanoids, diarylpentanoids, phenylpropenes, terpenes, terpenoids, sterols, alkaloids, and curcuminoids (mostly curcumin) [58]. The phenolic compounds in ginger are mainly gingerols, shogaols, and paradols, along with quercetin, zingerone, gingerenone A, and 6-dehydrogingerdione [59]. The main polyphenols reported in cocoa beans are catechins, which constitute about 37% of the polyphenol content, anthocyanidins (about 4%), and proanthocyanidins (about 58%) [60]. Green tea contains flavanols (mostly catechins), flavandiols, flavonoids, and phenolic acids which together contribute up to 30% of the dry weight [61]. Similar to investigations of fruit and vegetables, the differing levels of these phenolic compounds in the condiment samples tested are the most likely reason for the different AOX capacities.

Based on the recommendations in Apak et al. [11], we investigated dichloromethane and pure ethanol extractions of the capsicum, tomato, and pumpkin samples to understand the contributions from specific carotenoid molecules which are known to be insoluble in other solvent combinations. The dichloromethane and pure ethanol extracts were not fully compatible (stability of radical species [11] and turbidity issues [3, 8, 9, 12]) with ABTS and FC-TPC assays restricting our investigation to CUPRAC results alone (data not shown) and were therefore removed from any further comparison.

### 3.4. Assay Results—Comparison Within the Laboratory

Though the comparison of assay results in Table 2 shows some similarity with the published literature values, the validity of such comparisons remains questionable on the grounds of the recommendations presented in the IUPAC Technical report by Apak et al. [2], i.e., “results in any publications have to be carefully scrutinised as they may not be comparable as each AOX assay has a different mechanism, redox potential, reaction media, extracting solvent, etc. However, in one laboratory the results within one test system can be used for a ranking.” Based on this statement, the average results for each of the samples tested in the current study, utilising three independent assay systems (ABTS, CUPRAC, and TPC), were compared within each solvent system selected for the extractions (Milli-Q water, 40% ethanol, and 40% ethanol with m*β*CD).

The mutual correlation of these three assays was also analysed, and the results are given in Tables 3(a) and 3(b). The samples showed strong correlations between assay systems with *R*^2^ values as shown and further confirm the reliability and acceptance of these assay systems. A similar observation was described by Sariburun et al. [62] who noted a high positive correlation between the ABTS, CUPRAC, and TPC assays for water and methanol extracts of raspberry and blackberry cultivars and attributed that the AOX activity of fruit tested appears to be largely influenced by the phenolic compounds.

**Figure 6:**
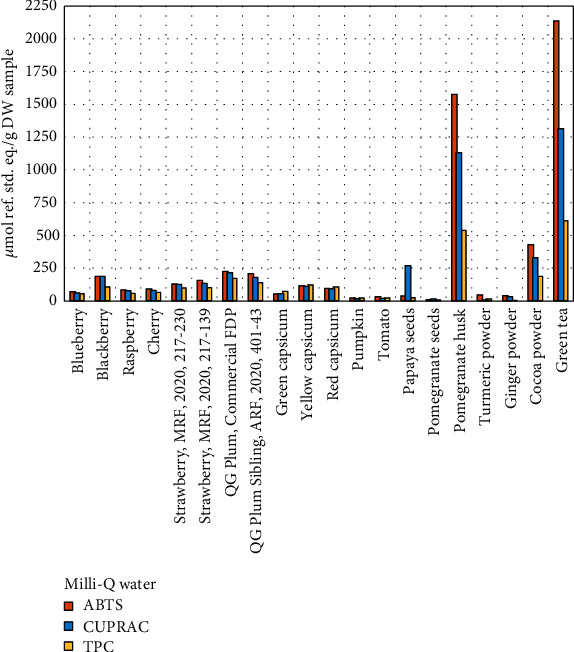
Comparison of ABTS, CUPRAC, and TPC assays in Milli-Q water.

**Figure 7:**
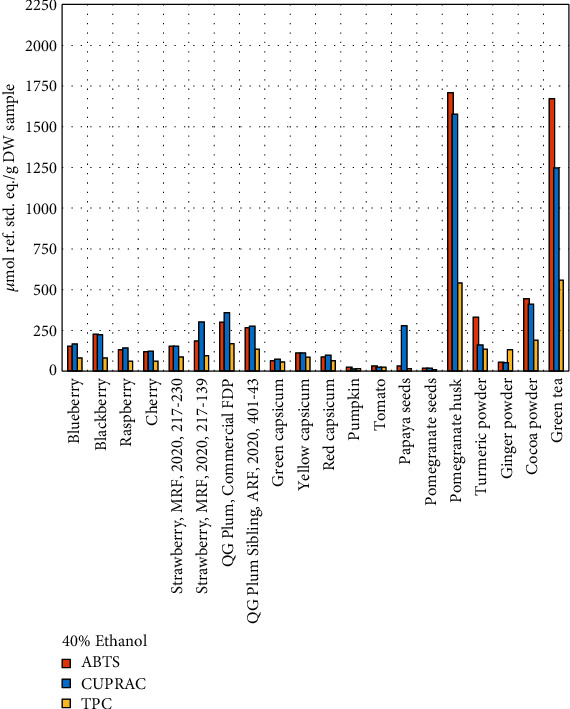
Comparison of ABTS, CUPRAC, and TPC assays in 40% ethanol.

**Figure 8:**
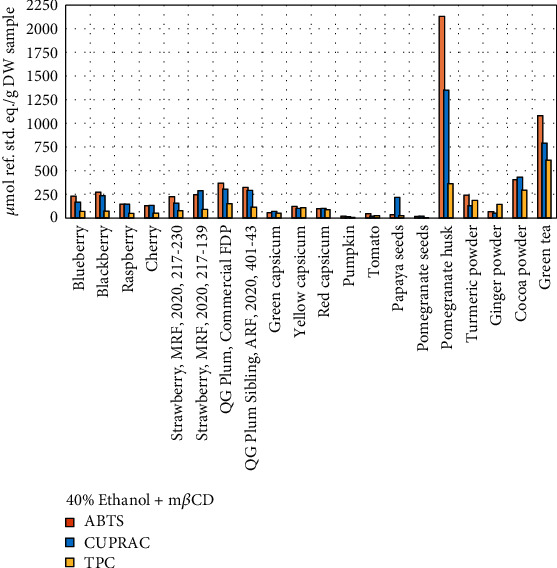
Comparison of ABTS, CUPRAC, and TPC assays in 40% ethanol modified with m*β*CD.

It is evident from the comparative graphs (Figures 6–8) that, in terms of their AOX capacity and TPC content, all the samples exhibited a similar trend and maintained their relative ranking for all three assays, regardless of the solvent system. This confirms the reliability and practical usefulness of these assays to be employed in any laboratory to introduce an internal ranking system for similar samples based on their AOX capacity and TPC assays.

## 4. Conclusions

- The current study employed two AOX capacity assays (ABTS and CUPARC) along with a FC-TPC assay, utilising both hydrophilic and lipophilic solvent systems, to measure the AOX properties of selected fruit, fruit waste, vegetables, and spices of commercial importance in Australia and worldwide. The assays were also verified within the laboratory to confirm their suitability
- The modified ABTS assay (at pH 4.5) and the modified FC-TPC assay (in 40% ethanol) used in this study were applied for the first time to a range of fruit, vegetable, and spice samples and compared the results from three different solvent systems
- The results obtained by this study utilising three independent assays in three different solvent systems exhibited good mutual correlation, confirming that the results are suitable to generate a reliable ranking system within the laboratory for similar sample matrices. This valuable information also enhances the usefulness of these assays
- It is evident from the results obtained that the use of both hydrophilic and lipophilic solvents for extractions is vital and must be incorporated into any AOX capacity and/or TPC assay, as the solvents can produce widely differing results depending upon the properties of the polyphenolic molecules present in the sample matrices
- Comparing the results of the current study with other published values proved problematic due to the vast differences in extraction solvents, assay systems, and the units of presentation presented in the literature. Even after converting the assay value units and applying the appropriate correction factors for moisture contents where appropriate, the current study revealed how challenging it is to make meaningful comparisons with previously reported AOX results
- The unique approach employed in this study provided a strong platform to build an internal reference database for the food commodities commonly consumed and of commercial importance in Australia and worldwide. There is potential to broaden the scope and further develop the reference database to include an increased range of sample matrices

## Figures and Tables

**Table 1:** Extraction solvents as presented in the references.

Reference	Samples tested	Extraction solvents
2	Green tea	Steeping in boiling water
16	Blueberry, blackberry, raspberry, strawberry	Not given
18	Blueberry	Acidified methanol
19	Blueberry, blackberry, raspberry	Aqueous methanol-acetone extraction followed by evaporation, reextraction in diethyl ether-ethyl acetate, final evaporation, and redissolution in methanol
20	Blueberry, blackberry, raspberry	Multiple extractions using methanol
21	Blueberry, blackberry, raspberry, cherry, strawberry	50 : 50 methanol-water extraction followed by 70 : 30 acetone-water extraction
22	Blueberry, blackberry, strawberry	80% methanol
23	Blueberry	60 : 40 ethanol-water
24	Raspberry, strawberry, plum, tomato	Methanol, n-hexane, varying solvents
25	Plum	Multiple extractions using 90% methanol
26	Plum	Methanolic extraction followed by 80% methanol, evaporation, and redissolution in methanol
29	Pumpkin	Multiple extractions using 80% methanol
30	Pumpkin	Various solvents, solvent combinations, and conditions
32	Tomato	Multiple extractions using 75% methanol in water
33	Tomato, capsicum	Multiple extractions using 75% methanol in water
34	Capsicum	Multiple extractions using various solvents and conditions
35	Papaya seeds	Absolute methanol and 80 : 20 methanol-water
36	Papaya seeds	Subcritical water extraction and conventional soxhlet extraction using water
37	Pomegranate husks	Multiple extractions using 75% aqueous methanol including 0.1% (*v*/*v*) formic acid
38	Pomegranate husks	Compared many different extractions solvents, their combinations and conditions
39	Papaya seeds	Multiple extractions of 95% ethanolic extract after partitioning with water, ethanol extract with petroleum ether, and ethyl acetate
40	Pomegranate husks	Decoction in boiling water
41	Pomegranate seeds, pomegranate husks	Multiple extractions using 80% methanol in water, evaporated and separate hydrolysis followed by extraction with 100% methanol containing 0.1% formic acid
42	Pomegranate husks	96% ethanol
47	Turmeric powder	Boiling water
48	Turmeric powder, ginger powder	Water and 50% ethanol-water at various conditions
49	Turmeric powder	Methanol, water, and ethanol
50	Turmeric	Expressed juice
51	Turmeric powder	Separate multiple extractions using 80% ethanol, 80% methanol, 80% acetone, and water
52	Turmeric powder	Dimethyl formamide
53	Ginger powder	80% methanol
54	Ginger powder	Separate extractions using 80% ethanol and water followed by solvent removal
56	Ginger powder	Multiple extractions using methanol followed by evaporation and redissolution in methanol
57	Cocoa powder	Separate extractions using water and 70% methanol after removing fat with n-hexane
58	Green tea	98°C boiling water
59	Green tea	Steeping in hot water at different temperatures
60	Green tea	Steeping in boiling water
61	Green tea	Steeping in freshly boiled water
62	Green tea	Multiple extractions using 80% methanol

**Table 2:** Range of AOX and TPC values of the samples in different solvent systems in comparison with the reported values after unit conversions.

Sample	Solvent	ABTS *μ*mol AA E/g DW	*^ɸ^* ABTS *μ*mol AAE/g DW (^∗^reference)	CUPRAC *μ*mol TE/g DW	*^ɸ^* CUPRAC *μ*mol TE/g DW (^∗^reference)	FC-TPC *μ*mol GAE/g DW	*^ɸ^* FC-TPC *μ*mol GAE/g DW (^∗^reference)
Blueberry	Milli-Q water	65.7-73.3	134-231 [14] 370.8 [15] 269.8 [16] 67.9 [17] 212.9 [18] 180.4 [19]	56.8-62.2	100-120 [20]	50.9-56.1	9.3-163.7 [21] 107-272 [14] 84.1 [15] 114.9 [16] 145.9 [17] 55.5 [18] 143-179 [20] 179-206 [20]
	40% ethanol	131.1-157.9		148.3-168.5		72.4-85.4	
	m*ß*CD	209.2-240.9		152.0-172.4		57.9-73.5	

Blackberry	Milli-Q water	174.3-192.2	683.1 [15] 225.4 [16] 155.6 [17] 163.1 [18]	157.9-204.5	_	96.2-114.3	2.9-40.6 [21] 106.5 [15] 99.9 [16] 413.9 [17] 32.8 [18]
	40% ethanol	207.6-228.6		197.6-233.6		70.4-89.9	
	m*ß*CD	248.4-298.4		197.6-257.5		61.7-84.1	

Raspberry	Milli-Q water	75.4-92.1	472.3 [15] 297.5 [16] 8.2 [17]	60.9-86.5	184 [22]	50.2-62.2	6.7-107.1 [21] 34.0 [15] 118.4 [16] 184.5 [17] 89 [22]
	40% ethanol	112.5-142.4		118.3-153.0		54.0-63.7	
	m*ß*CD	124.6-162.1		118.7-169.6		42.3-55.4	

Cherry	Milli-Q water	80.2-96.4	92.5 [17]	65.2-94.2	_	56.3-70.1	136.2 [17]
	40% ethanol	94.7-135.7		109.4-126.3		52.7-67.7	
	m*ß*CD	108.9-141.4		113.3-135.8		36.9-57.7	

Strawberry; MRF, 2020, 217-230	Milli-Q water	121.6-130.2	153.2 [17] 63.1 [18] 103.5 [19]	106.1-140.4	259 [22]	95.0-99.4	18.6-42.1 [21] 500.8 [17] 16.0 [18] 129 [22]
	40% ethanol	147.4-149.2		144.6-153.0		82.8-90.2	
	m*ß*CD	210.3-225.0		146.5-155.4		70.3-79.1	
Strawberry; MRF, 2020, 2017-139	Milli-Q water	138.6-163.8		130.5-131.8		89.4-111.5	
	40% ethanol	176.4-183.6		283.7-304.8		89.1-99.8	
	m*ß*CD	235.5-243.5		259.7-289.8		86.7-91.9	

Queen Garnet plum commercial FDP	Milli-Q water	215.2-223.1	140.8 [19]	201.0-220.2	182 [16] 65-206 [23]	164.9-171.4	121 [22] 25-62 [23]
	40% ethanol	286.1-296.1		336.4-364.3		163.3-165.2	
	m*ß*CD	352.3-366.2		265.2-334.6		144.6-148.5	
Queen Garnet plum sibling ARF, 2020, 401-43	Milli-Q water	199.5-211.1		175.7-180.5		133.8-140.9	
	40% ethanol	257.8-267.2		267.1-273.9		131.5-134.0	
	m*ß*CD	314.2-325.3		274.6-303.7		113.4-115.6	

Green capsicum	Milli-Q water	41.1-62.5	29.4-295 [24]	35.2-74.2	93.6-98.8 [25] 64.8-188.4 [24] 65.7 [25]	61.8-86.0	63.7-94.5 [25] 23.5-130.4 [24]
	40% ethanol	55.8-69.1		55.8-89.4		47.6-65.1	
	m*ß*CD	47.1-65.6		46.7-91.7		42.8-62.2	
Yellow capsicum	Milli-Q water	103.3-117.7		101.8-110.1		114.2-128.1	
	40% ethanol	100.8-110.6		95.1-114.8		74.6-89.8	
	m*ß*CD	114.2-123.1		86.1-106.5		101.8-109.1	
Red capsicum	Milli-Q water	88.4-99.7		84.1-100.9		97.8-105.6	
	40% ethanol	77.9-92.1		76.1-107.2		51.8-73.6	
	m*ß*CD	79.3-111.9		89.2-108.4		67.4-99.8	

Pumpkin	Milli-Q water	19.7-26.2	4.5-7.2 [26] 3.2-4.19 [26]	11.2-24.1	3.97-10.25 [27]	19.6-27.4	5.49-13.98 [26] 5.48-10.98 [26]
	40% ethanol	21.5-26.0		11.2-18.3		12.9-17.5	
	m*ß*CD	18.4-23.1		9.1-19.0		5.8-8.9	

Tomato	Milli-Q water	30.8-33.2	24.5 [28]	22.9-23.6	16.6 [28]	22.1-25.0	27.8 [25] 27.1 [22] 16.1 [28]
	40% ethanol	32.0-33.6		19.8-20.3		22.2-23.8	
	m*ß*CD	45.3-48.0		19.3-19.9		22.1-24.7	

Papaya seeds	Milli-Q water	35.5-41.1	_	267.7-272.8	_	24.0-27.1	56.5-426.5 [29, 30] 16.3 [31] 66.5 [31]
	40% ethanol	32.1-34.0		277.9-283.0		15.8-16.8	
	m*ß*CD	33.4-33.9		207.8-226.5		21.3-26.2	

Pomegranate seeds	Milli-Q water	10.6-11.0	_	13.7-19.5	239 [32]	3.0-9.5	82 [32]
	40% ethanol	16.8-17.8		17.0-18.6		9.1-9.4	
	m*ß*CD	15.3-16.4		17.2-20.3		4.5-4.8	

Pomegranate husks	Milli-Q water	1381.9-1502.6	_	936.4-1126.3	2080-8090 [33] 1118 [32] 663-3428 [34] 3756 [35]	477.2-517.3	623-1545 [33] 423 [32] 175-1060 [34] 497 [36]
	40% ethanol	1532.8-1636.4		1440.2-1493.0		433.5-578.9	
	m*ß*CD	1905.8-2035.2		1230.2-1267.5		314.9-372.5	

Turmeric powder	Milli-Q water	45.3-49.1	_	7.6-7.8	600-800 [37]	15.8-16.9	24.6 [38] 4.5 [39] 39.9 [40] 4.0 [41] 1011.6 [42] 22.3 [42]
	40% ethanol	327.0-334.2		154.6-166.9		132.7-137.8	
	m*ß*CD	124.5-136.8		124.5-136.8		173.1-207.4	

Ginger powder	Milli-Q water	37.7-46.9	130.6-398.6 [43]	32.2-34.7	229.3-487.0 [43]	5.6-7.7	490.2-794.8 [43] 310.6-808.5 [44] 13.5-99.2 [45] 4.0 [39]
	40% ethanol	50.7-57.3		48.2-54.4		119.2-152.4	
	m*ß*CD	64.9-65.1		49.5-58.8		133.6-152.6	

Cocoa powder	Milli-Q water	425.5-436.6	_	328.8-333.9	_	184.2-191.5	17-199 [46]
	40% ethanol	437.8-448.7		403.7-421.1		189.3-193.4	
	m*ß*CD	385.8-422.7		414.5-450.0		292.0-301.1	

Green tea	Milli-Q water	2127.3-2148.8	_	1305.0-1326.0	1000 [2] 50-4410 [47] 1010 [48]	609.8-618.5	870-1481.3 [49] 305.7 - 687.7 [50] 336.6-605.5 [51]
	40% ethanol	1671.6-1675.3		1240.3-1257.9		555.6-560.7	
	mßCD	1029.8-1122.2		772.2-813.4		579.1-630.1	

*^ɸ^* Reported values after unit conversions. ^∗^Reference.

**(a):** 

		MQ-water			40% ethanol			m*ß*CD		
		ABTS	CUPRAC	TPC	ABTS	CUPRAC	TPC	ABTS	CUPRAC	TPC
MQ-water	ABTS	1.0000								
	CUPRAC	0.9852	1.0000							
	TPC	0.9779	0.9742	1.0000						

40% ethanol	ABTS	0.9758	0.9701	0.9677	1.0000					
	CUPRAC	0.9411	0.9679	0.9526	0.9773	1.0000				
	TPC	0.9634	0.9511	0.9615	0.9844	0.9571	1.0000			

m*ß*CD	ABTS	0.8578	0.8813	0.8887	0.9376	0.9634	0.9154	1.0000		
	CUPRAC	0.8727	0.9127	0.9075	0.9376	0.9822	0.9172	0.9815	1.0000	
	TPC	0.9230	0.8900	0.9011	0.9065	0.8428	0.9402	0.7463	0.7761	1.0000

**(b):** 

		MQ-water			40% ethanol			m*ß*CD		
		ABTS	CUPRAC	TPC	ABTS	CUPRAC	TPC	ABTS	CUPRAC	TPC
MQ-water	ABTS	1.0000								
	CUPRAC	0.7953	1.0000							
	TPC	0.8892	0.7215	1.0000						

40% ethanol	ABTS	0.8309	0.5649	0.6864	1.0000					
	CUPRAC	0.8138	0.8834	0.7282	0.7835	1.0000				
	TPC	0.7514	0.4628	0.6529	0.8632	0.6691	1.0000			

m*ß*CD	ABTS	0.8492	0.6086	0.7806	0.9399	0.8286	0.8419	1.0000		
	CUPRAC	0.8844	0.8767	0.7774	0.8202	0.9825	0.6999	0.8708	1.0000	
	TPC	0.7628	0.4862	0.5845	0.8551	0.6219	0.9277	0.7346	0.6566	1.0000

*n* = 20. ^∗^The values in (b) are the correlations taken (*n* = 18) without considering green tea and pomegranate husk sample results due to the limited sample numbers and their very high assay results.

**Table 4:** Moisture content of the freeze-dried/oven-dried/fresh samples analysed.

Sample	Sample condition	Moisture content (g/100g)
Blueberry	Freeze-dried powder	4.0
Blackberry	Freeze-dried powder	3.2
Raspberry	Freeze-dried powder	3.5
Cherry	Freeze-dried powder	3.2
Strawberry; MRF, 2020, 217-230	Freeze-dried powder	3.1
Strawberry; MRF, 2020, 2017-139	Freeze-dried powder	2.8
Queen Garnet plum; commercial FDP	Dry powder ^∗^	2.6
Queen Garnet plum sibling; ARF, 2020, 401-43	Freeze dried powder	1.5
Green capsicum	Freeze dried powder	2.5
Yellow capsicum	Freeze dried powder	4.1
Red capsicum	Freeze dried powder	4.9
Pumpkin	Freeze dried powder	3.1
Tomato	Fresh puree	93.5
Papaya seeds	Oven-dried powder	4.2
Pomegranate seeds	Oven-dried powder	4.1
Pomegranate husk	Oven-dried powder	7.4
Turmeric powder	Dry powder ^∗^	7.7
Ginger powder	Dry powder ^∗^	10.5
Cocoa powder	Dry powder ^∗^	6.0
Green tea	Dry leaves^∗^	4.6

^∗^Commercial sample used as received.

**Table 5:** Slope of calibration curves (from Figure 4) in different solvents at different time points and at different wavelengths.

Assay solvent and time	750 nm	760 nm
FC-TPC assay in 40% ethanol; 20 min	*y* = 0.010200*x* *R*^2^ = 0.999822	*y* = 0.010088*x* *R*^2^ = 0.999826

FC-TPC assay in 40% ethanol; 90 min	*y* = 0.009841*x* *R*^2^ = 0.999763	*y* = 0.009770*x* *R*^2^ = 0.999709

FC-TPC assay in Milli-Q water; 20 min	*y* = 0.008917*x* *R*^2^ = 0.998418	*y* = 0.008956*x* *R*^2^ = 0.998708

FC-TPC assay in Milli-Q water; 90 min	*y* = 0.004062*x* *R*^2^ = 0.999916	*y* = 0.004053*x* *R*^2^ = 0.999901

## Data Availability

The data used to support the findings of this study are included within the article.
